# Supplementary material for: Effectiveness of physical therapy for locomotive syndrome: A systematic review and meta-analysis protocol
Source: PLoS One. 2025 Aug 14;20(8):e0329758. doi: 10.1371/journal.pone.0329758 (PMC12352771; doi:10.1371/journal.pone.0329758)
Supplement: S2 Appendix — (DOCX) [file pone.0329758.s002.docx]

**Supplementary appendix 1**

Search strategy

(1) PubMed

| Search number | Query |
| --- | --- |
| 1 | locomot*-syndrome*[tiab] |
| 2 | Rehabilitation[mh] OR Rehabilitation[sh] OR rehab*[tiab] OR habili*[tiab] OR neurorehab*[tiab] OR telerehab*[tiab] |
| 3 | Physical Therapy Modalities[mh] OR physical-ther*[tiab] OR physiother*[tiab] OR physio-ther*[tiab] OR physiatr*[tiab] OR neurophysiother*[tiab] OR motion*-ther*[tiab] OR manipulat*[tiab] OR manual-ther*[tiab] OR "manual therapy"[tiab:~2] OR manual-treatment*[tiab] OR myofunctional-treatment*[tiab] OR "myofunctional therapy"[tiab:~3] OR "myofunctional therapies"[tiab:~3] OR stretch*[tiab] OR massag*[tiab] OR mobilization[tiab] |
| 4 | Exercise[mh] OR exercis*[tiab] OR training*[tiab] OR kinesither*[tiab] OR kinesi-ther*[tiab] OR aerobic*[tiab] OR hydrother*[tiab] OR hydro-ther*[tiab] OR hydrotreat*[tiab] OR hydro-treat*[tiab] OR aquatic*[tiab] OR ai-chi[tiab] OR pool-ther*[tiab] OR pool-treat*[tiab] OR tai-chi[tiab] |
| 5 | Electrical Stimulation[mh] OR electric*-stimulat*[tiab] OR "electric stimulation"[tiab:~2] OR "electrical stimulation"[tiab:~2] OR electricalstimulat*[tiab] OR EMS[tiab] OR NMS[tiab] OR NMES[tiab] |
| 6 | #2 OR #3 OR #4 OR #5 |
| 7 | #1 AND #6 |
| 8 | (Controlled Clinical Trial[pt] OR randomized[tiab] OR randomised[tiab] OR placebo[tiab] OR randomly[tiab] OR trial[ti] OR Epidemiologic Study Characteristics[mh] OR Observational Study[pt] OR case-control*[tiab] OR retrospective[tiab] OR longitudinal[tiab] OR cohort*[tiab] OR prospective[tiab] OR observatio*-stud*[tiab] OR "observational-study"[tiab:~2] OR "observational-studies"[tiab:~2] OR followup[tiab] OR follow-up[tiab] OR cross-sectional[tiab] OR pilot*[tiab] OR group*[tiab]) NOT (Animals[mh] NOT Humans [mh]) |
| 9 | #7 AND #8 |
| 10 | eng[la] OR jpn[la] OR tha[la] |
| 11 | #9 AND #10 |

(2) Cochrane Central Register of Controlled Trials

| Search number | Query |
| --- | --- |
| 1 | (locomot* NEXT syndrome*):ti,ab,kw |
| 2 | [mh Rehabilitation] OR (rehab* OR habili* OR neurorehab* OR telerehab*):ti,ab,kw |
| 3 | [mh "Physical Therapy Modalities"] OR (physical-ther* OR physiother* OR physio-ther* OR physiatr* OR neurophysiother* OR (motion* NEXT ther*) OR manipulat* OR (manual NEAR/3 ther*) OR manual-treatment* OR myofunctional-treatment* OR (myofunctional NEAR/4 ther*) OR stretch* OR massag* OR mobilization):ti,ab,kw |
| 4 | [mh Exercise] OR (exercis* OR training* OR kinesither* OR kinesi-ther* OR aerobic* OR hydrother* OR hydro-ther* OR hydrotreat* OR hydro-treat* OR aquatic* OR ai-chi OR pool-ther* OR pool-treat* OR tai-chi):ti,ab,kw |
| 5 | [mh "Electrical Stimulation"] OR (electric* NEAR/3 stimulat* OR electricalstimulat* OR EMS OR NMS OR NMES):ti,ab,kw |
| 6 | {OR #2-#5} |
| 7 | #1 AND #6 |
| 8 | Conference proceeding:pt |
| 9 | #7 NOT #8 |
| 10 | english:la OR japanese:la OR thai:la |
| 11 | #9 AND #10 |

(3) CINAHL

| Search number | Query |
| --- | --- |
| 1 | TI locomot*-syndrome* OR AB locomot*-syndrome* |
| 2 | MH Rehabilitation+ OR TI (rehab* OR habili* OR neurorehab* OR telerehab*) OR AB (rehab* OR habili* OR neurorehab* OR telerehab*) |
| 3 | TI (physical-ther* OR physiother* OR physio-ther* OR physiatr* OR neurophysiother* OR motion*-ther* OR manipulat* OR manual N2 ther* OR manual-treatment* OR myofunctional-treatment* OR myofunctional N3 ther* OR stretch* OR massag* OR mobilization) OR AB (physical-ther* OR physiother* OR physio-ther* OR physiatr* OR neurophysiother* OR motion*-ther* OR manipulat* OR manual N2 ther* OR manual-treatment* OR myofunctional-treatment* OR myofunctional N3 ther* OR stretch* OR massag* OR mobilization) |
| 4 | MH Exercise+ OR TI (exercis* OR training* OR kinesither* OR kinesi-ther* OR aerobic* OR hydrother* OR hydro-ther* OR hydrotreat* OR hydro-treat* OR aquatic* OR ai-chi OR pool-ther* OR pool-treat* OR tai-chi) OR AB (exercis* OR training* OR kinesither* OR kinesi-ther* OR aerobic* OR hydrother* OR hydro-ther* OR hydrotreat* OR hydro-treat* OR aquatic* OR ai-chi OR pool-ther* OR pool-treat* OR tai-chi) |
| 5 | MH Electric Stimulation+ OR TI (electric* N2 stimulat* OR electricalstimulat* OR EMS OR NMS OR NMES) OR AB (electric* N2 stimulat* OR electricalstimulat* OR EMS OR NMS OR NMES) |
| 6 | S2 OR S3 OR S4 OR S5 |
| 7 | S1 AND S6 |
| 8 | (MH Clinical Trials+ OR TI (randomised OR randomized OR placebo OR randomly OR trial) OR AB (randomised OR randomized OR placebo OR randomly) OR MH Clinical Trial Registry OR MH Epidemiological Research+ OR TI (case-control* OR retrospective OR longitudinal OR cohort* OR prospective OR observatio* N2 stud* OR followup OR follow-up OR cross-sectional OR pilot* OR group*) OR AB (case-control* OR retrospective OR longitudinal OR cohort* OR prospective OR observatio* N2 stud* OR followup OR follow-up OR cross-sectional OR pilot* OR group*)) NOT (MH (Animals+ NOT Human)) |
| 9 | S7 AND S8 |
| 10 | LA (english OR japanese OR thai) |
| 11 | S9 AND S10 |

(4) PEDro

| Search number | Query |
| --- | --- |
| 1 | "locomotive syndrome" |

(5) Scopus

| Search number | Query |
| --- | --- |
| 1 | TITLE-ABS-KEY(locomot*-syndrome*) |
| 2 | TITLE-ABS-KEY(rehab* OR habili* OR neurorehab* OR telerehab*) |
| 3 | TITLE-ABS-KEY(physical-ther* OR physiother* OR physio-ther* OR physiatr* OR neurophysiother* OR motion*-ther* OR manipulat* OR manual-ther* OR (manual W/2 ther*) OR manual-treatment* OR myofunctional-treatment* OR (myofunctional W/3 ther*) OR stretch* OR massag* OR mobilization) |
| 4 | TITLE-ABS-KEY(exercis* OR training* OR kinesither* OR kinesi-ther* OR aerobic* OR hydrother* OR hydro-ther* OR hydrotreat* OR hydro-treat* OR aquatic* OR ai-chi OR pool-ther* OR pool-treat* OR tai-chi) |
| 5 | TITLE-ABS-KEY((electric* W/2 stimulat*) OR electricalstimulat* OR {EMS} OR {NMS} OR {NMES}) |
| 6 | #2 OR #3 OR #4 OR #5 |
| 7 | #1 AND #6 |
| 8 | (INDEXTERMS("Controlled Clinical Trial" OR "Randomized Controlled Trial" OR "Clinical Trials as Topic") OR TITLE-ABS-KEY(randomi?ed OR placebo OR randomly) OR TITLE(trial) OR TITLE-ABS-KEY((epidemiologic* W/2 stud*) OR case-control* OR retrospective OR longitudinal OR cohort* OR prospective OR (observatio* W/2 stud*) OR followup OR follow-up OR cross-sectional OR pilot* OR group*)) AND NOT (INDEXTERMS(animal* AND NOT human*)) |
| 9 | #7 AND #8 |
| 10 | LANGUAGE(english OR japanese OR thai) |
| 11 | #9 AND #10 |

(6) Ichushi

| Search number | Query |
| --- | --- |
| 1 | ロコモティブシンドローム/TH or ロコモ/TA or "locomotive syndrome"/TA |
| 2 | リハビリテーション/TH or リハビリ/TA or ハビリテーション/TA |
| 3 | 理学療法/TH or 理学療法/TA or 物理療法/TA or フィジカルセラピー/TA or フィジオセラピー/TA or モーションセラピー/TA or マニピュレーション/TA or 徒手/TA or マニュアルセラピー/TA or 筋機能療法/TA or マイオファンクショナルセラピー/TA or ストレッチ/TA or マッサージ/TA or モビライゼーション/TA or モビリゼーション/TA |
| 4 | 身体運動/TH or 身体運動/TA or 運動療法/TA or 運動習慣/TA or 運動介入/TA or 体操/TA or 訓練/TA or 機能療法/TA or エクササイズ/TA or トレーニング/TA or エアロビ/TA or 水治療/TA or 水中運動/TA or 水中訓練/TA or 水中歩行/TA or 運動浴/TA or 浴中運動/TA or プール治療/TA or プール療法/TA or ロコトレ/TA |
| 5 | 電気刺激/TH or 電気刺激/TA or 電気的刺激/TA or EMS/TA or NMES/TA |
| 6 | #2 or #3 or #4 or #5 |
| 7 | #1 and #6 |
| 8 | (臨床試験/TH or ランダム化比較試験/RD or 準ランダム化比較試験/RD or ランダム/TA or プラセボ/TA or 疫学的研究デザイン/TH or 臨床研究・疫学研究/TH or 疫学研究/TA or 症例対照/TA or ケースコントロール/TA or 後ろ向き/TA or 後向き/TA or レトロスペクティブ/TA or 縦断研究/TA or コホート/TA or 前向き/TA or プロスペクティブ/TA or 観察研究/TA or 追跡/TA or 横断研究/TA or 横断的研究/TA or パイロット/TA or グループ/TA) not (動物/CK not ヒト/CK) |
| 9 | #7 and #8 |
| 10 | PT=会議録,座談会,レター,コメント,一般 |
| 11 | #9 not #10 |

(7) Thai-Journal Citation Index Centre

| Search number | Query |
| --- | --- |
| 1 | locomotive syndrome　[Article Title] OR locomotive syndrome　[Abstract] OR  locomotive syndrome　[Keyword] |
